# Age trends of genetic parameters, early selection and family by site interactions for growth traits in *Larix kaempferi* open-pollinated families

**DOI:** 10.1186/s12863-016-0400-7

**Published:** 2016-07-07

**Authors:** Shu Diao, Yimei Hou, Yunhui Xie, Xiaomei Sun

**Affiliations:** State Key Laboratory of Tree Genetics and Breeding, Research Institute of Forestry, Chinese Academy of Forestry, Beijing, 100091 China; Key Laboratory of Tree Breeding and Cultivation of State Forestry Administration, Research Institute of Forestry, Chinese Academy of Forestry, Beijing, 100091 China; Research Institute of Forestry Science, Jianshi County, Hubei Province 445300 China

**Keywords:** Heritability, Early selection, Family by site interactions, Growth traits

## Abstract

**Background:**

Japanese larch (*Larix kaempferi*) as a successful exotic species has become one of the most important economic and ecological conifers in China. In order to broaden the genetic resource of *Larix kaempferi*, an effort was made in 1996 to introduce 128 families from seven seed orchards in Japan, with which to establish two progeny trials in climatically different environments. The experiment was aimed to determine the strategy of early selection, particularly important for long-rotated Japanese larch, and the optimal breeding program for specific environments.

**Result:**

Growth trajectories revealed different growth performances of stem height (HGT) and diameter at breast height (DBH) in two different environments, Hubei and Liaoning. In both sites, there were marked variabilities in HGT, DBH and volume (VOL) among families at each year. The trends of individual and family heritability and age-age correlations were found to follow a certain dynamic pattern. Based on these trends, the optimum selection age was determined at four years for HGT and five years for DBH in Hubei and Liaoning. Genetic gains for VOL were 34.4 and 6.04 % in Hubei and Liaoning respectively when selection ratio was 10 % at age 16. Type-B correlations were less than 0.67 and rank correlations of breeding value were less than 0.4 for HGT, DBH and VOL between the two sites, revealing that there exist pronounced family-by-site interactions for the growth traits of *Larix kaempferi*.

**Conclusions:**

Early selection for *Larix kaempferi* is an effective strategy to overcome its long rotation age. In early selection, dual growth trait selection is more effective than single one. Regionalization deployment should be considered in *Larix. kaempferi* breeding program based on different environmental factors.

**Electronic supplementary material:**

The online version of this article (doi:10.1186/s12863-016-0400-7) contains supplementary material, which is available to authorized users.

## Background

Japanese larch (*Larix kaempferi*), one successfully exotic species in China, is becoming the preferred coniferous species for plantation due to its superior performance of genotype on fast-growing at early ages, wood properties, pest resistance and wide adaptation, better than native larch, such as *L. principis-rupprechtii* and *L.olgensis* [1]. *L. kaempferi*, also as one of important short-rotation forest species has bright market prospects for pulpwood plantation in north subtropical mountains in China [2]. Japanese larch has been introduced to China at the end of 19th century and the breeding programs for Japanese larch had been conducted for several decades in China [3]. In 1996, in order to broaden the genetic resource of *L. kaempferi* in China, 128 open-pollinated families from seven seed orchards and 84 natural stands originated from six provenances in Japan were introduced to China supported by Japan International Cooperation Agency (JICA), which provided a good foundation for improving breeding programs of *L.kaempferi* in China.

The major rotation age for *L. kaempferi* is about 25–28 years as pulpwood in China [4]. A strategy of early age selection for *L. kaempferi* is a wise choice in tree improvement programs and this would bring huge economic benefits. In a tree-breeding program, the accurate assessment of genetic parameters is critical for developing successful breeding strategies [5]. Based on the age trends of genetic parameters for growth traits, such as heritability and age-age correlations, the early selection age can be predicted. Few studies on *L. kaempferi* have been done for estimation of age-trends of genetic parameters for growth traits and for early selection [4, 6, 7], and the suggested optimum selection ages for Japanese larch has varied among different studies, i.e., 6 years for HGT and DBH in family selection and 8 years in individual-tree selection based on 47 open-pollinated families [6], 5 years for DBH and 2 years for HGT based on 78 clones [4] and 10 years for HGT based on 10 clones [7]. In these studies, the limited sample size and investigation years might have restricted the reliability and precision of genetic parameter estimates. Therefore, long term genetic experiments with large sample sizes are needed to be conducted.

Genotype by environment interaction (GEI) refers to the phenomenon that the genotypes which are superior in one environment may not be correspondingly superior elsewhere [8]. Regionalization deployment becomes an important issue in tree breeding program, in which tree breeder should consider to balance the relative costs and benefits of regionalization [9]. The importance of genotype by site interaction for growth traits has been assessed in several conifer species, including *Pinus radiata* [10–12], *P. taeda* [13], *P. pinaster* [14, 15] and *Pic*ea *abies* [16]. However, some other researches also reported that although family by site interaction existed, regionalization is not considerable because of more economic costs and little genetic gain [17, 18]. Significant family by site interaction has been reported in the *L.kaempferi* progeny tests in Japan [19, 20], which indicates that the genotype by environment interaction is not neglectable to realize maximum genetic gain for Japanese larch. However, studies about family by site interaction were seldom reported for *L. kaempferi* in China*.* More attention should be given to genotype by environment interactions for optimal organization of breeding and deployment programs in China.

Two plantations of *L. kaempferi* established in Liaoning and Hubei were involved in our research including 132 families in Hubei and 78 families in Liaoning. The objectives of this study were: (1) to determine the age trends of heritabilities and age-age correlations for *L. kaempferi*; (2) to evaluate the optimum selection age for *L. kaempferi*; (3) to estimate the family by site interaction of *L. kaempferi* between Hubei and Liaoning.

## Methods

### Plant materials

The data were collected from two 16–year–old progeny trials in Jianshi County, Hubei Province and Qingyuan County, Liaoning Province. The natural conditions of these two experimental plantations were shown in Table 1. These two experimental plantations were established in 1998 based on JICA project. 132 families (128 originated from Japan, four controls originated from China) were planted in the experimental trial in Hubei and 78 of 132 families (including four controls from China) were also planted in the experimental trial in Liaoning. The detailed origin and natural conditions of these families were shown in Table 2. The field design was essentially randomized complete blocks with eight-tree two-row plots and six replicates. At the beginning of our investigation, 3578 individuals were included in Hubei trial and 1402 individuals were included in Liaoning trial. In the last investigation in 2013, 2475 and 922 individuals were respectively remained in these two trials due to death and thinning. All the families were reserved in Hubei and 76 families were remained in Liaoning at the last investigation. The thinning was done at age 10 in Hubei reserving all the families under breeder’s guide and at age 14 in Liaoning for the production under no breeder’s guide.

**Table 1:** The natural conditions of two progeny trials.

	Hubei	Liaoning
Latitude	30° 48'	42° 02'
Longitude	110° 3'	124° 50'
Elevation (m)	1600–1800	300
Mean annual rainfall (mm)	1500–1800	780
Soil type	Yellow brown soil of mountain	Brown forest soil of mountain
Mean annual temperature	9 °C	4.6 °C
Extremely low temperature	−10 °C	−37.6 °C
Extremely high temperature	29 °C	36.5 °C
The climatic type	Northern subtropical zone	Continental monsoon climate
Annual accumulated temperature ≧10 °C (°C)	2200–2300	2720.3
Annual sunshine hours(h)	1532.9	2433

**Table 2:** The origin and natural conditions of 132 families planted in progeny tests trials. The S and P in parentheses represent source of seeds and parent stand respectively

Order of families	Origin	Source of seeds (S) or parent stand (P)	The state of seed collection sites				
			Latitude	Longitude	Altitude (m)	Annual average temperature (°C)	Mean annual rainfall (mm)
85–88	Japan	Bibai in Hokkaido (S)	43° 20'	141° 50'	20	7	1305
89–118	Japan	Kuriyama in Hokkaido (S)	43° 08'	141° 45'	26	7.2	1238
198–205							
207–213							
119–125	Japan	Morioka in Iwate-ken (S)	39° 42'	141° 10'	135	10.3	1310
126–193	Japan	Komoro in Nagano (S)	36° 20'	138° 40'	843	9.9	1578
194	Japan	Engaru in Hokkaido (S)	44° 00'	143° 20'	236	5.3	929
195–196	Japan	Esashi in Iwate-ken (S)	39° 10'	141° 12'	40	10.7	1203
197	Japan	Azumino in Nagano (S)	36° 10'	137° 45'	1315	7.1	1913
214	China	Dandong in Liaoning (P)	40° 30'	124° 05'	320	8	750
215	China	Laoshan in Shandong (P)	36° 05'	120° 25'	110	12	1000
216	China	Qingyuan in Liaoning (S)	42° 45'	125° 48'	550	6	650
217	China	Jianshi in Hubei (P)	30° 45'	109° 45'	1700	15	1600

### Data collection

In Hubei, height (HGT) was measured at ages 1–4 years, and both HGT and diameter at breast (DBH) were measured at ages 5–8, 10, 11, 15 and 16 years. In Liaoning, HGT was measured at ages 1 and 2 years and both HGT and DBH were measured at ages 4–7 and 16 years. All data collections comply with national regulations. Individual tree volume (VOL in m^3^) was calculated using the following volume formula (1) in Hubei and (2) in Liaoning [21]:

1 $$ \mathrm{V}=0.00005108295689 \times {\mathrm{DBH}}^{1.857298121} \times {\mathrm{HGT}}^{1.017901505} $$

2 $$ \mathrm{V}=0.0000592372 \times {\mathrm{DBH}}^{1.8655726} \times {\mathrm{HGT}}^{0.98098926} $$

### Statistical analysis

A mixed linear model was used to analyze the variance components of the growth traits. The following model was used:

3 $$ {Y}_{ijk}=\mu + {B}_i + {f}_j + f{b}_{ij} + {e}_{ijk} $$

Where *Y*_*ijk*_ is the observation on the *k*^*th*^ tree in the *j*^*th*^ family in *i*^*th*^ replicate, *μ* is the overall mean, *B*_*i*_ is the fixed effect of *i*^*th*^ replicate, *f*_*j*_ is the random effect of *j*^*th*^ family which follows the Normally and independently Distribution NID (0, *σ*_*f*_^2^), *fb*_*ij*_ is the random interaction effect of the *i*^*th*^ replicate by the *k*^*th*^ family which follows the NID (0, *σ*_*fb*_^2^) and the *e*_*ijk*_ is random error within plot which follows the NID (0, *σ*_*e*_^2^).

The estimates of variance components for random effects of *u* were referred to *σ*_*f*_^2^, *σ*_*fb*_^2^ and *σ*_*e*_^2^, representing variance components of family, family by block interaction and residual error, respectively. The significance of the variance components were tested using the method of log-likelihood ratio tests [22].

The family heritability (*h*_*f*_^2^) and individual-tree heritability (*h*_*i*_^2^) were calculated using the following equations [23, 24]

4 $$ {h_f}^2={\sigma_f}^2/\ \left({\sigma_e}^2/r{n}_h+{\sigma_{fb}}^2/r+{\sigma_f}^2\right) $$

5 $$ {h_i}^2=4{\sigma_f}^2/\ \left({\sigma_e}^2+{\sigma_{fb}}^2+{\sigma_f}^2\right) $$

The number of trees in a plot is not eight due to death and thinning, leading to the unbalanced data. The numbers of trees were substituted by the adjusted numbers of trees in a plot n_h_ [25]:

6 $$ {n}_h=(bf)/{\displaystyle \sum_{i=1}^b}{\displaystyle \sum_{j=1}^f}\left(1/{n}_{ij}\right) $$

Where n_h_ is the adjusted number of trees in a plot, b is the number of replicates, f is the numbers of families and n_ij_ represents the numbers of individual trees of *j*^*th*^ families within *i*^*th*^ replicate.

The phenotypic variation coefficient (CVP) and genetic variation coefficient (CVG) were calculated using the following equations:

7 $$ CVG\ \left(\%\right)=\sqrt{{\sigma_f}^2}/\ \overline{X}\times 100 $$

8 $$ CVP\ \left(\%\right) = \sqrt{{\sigma_p}^2}/\ \overline{X}\times 100 $$

where *σ*_*p*_^2^ is phenotypic variance, *σ*_*f*_^2^ is family variance and ‾X is the overall mean of growth traits.

The phenotypic correlation and genetic correlation between traits (HGT or DBH) at early year with the corresponding traits or VOL at 16 years were calculated as the following Eqs. (9) and (10). The unstructured general covariance matrix (US) in Asreml was used in the analysis.

9 $$ rp=\sigma p(xy)/\sqrt{{\displaystyle {\sigma}_{p(x)}^2}\times {\displaystyle {\sigma}_{p(y)}^2}} $$

10 $$ rg=\sigma g(xy)/\sqrt{{\displaystyle {\sigma}_{g(x)}^2}\times {\displaystyle {\sigma}_{g(y)}^2}} $$

Where *rp* is phenotypic correlation, *rg* is genetic correlation; *σp*(*xy*) and *σg*(*xy*) represent respectively phenotypic and genetic covariance component between traits (HGT or DBH) at early year with the corresponding traits or VOL at 16 years; *σ*^2^_*p*(*x*)_ is phenotypic variance component for traits (HGT or DBH) at early years, *σ*^2^_*p*(*y*)_ is the phenotypic variance components for corresponding traits or VOL at 16 years; *σ*^2^_*g*(*x*)_ is genetic variance component for traits (HGT or DBH) at early years, *σ*^2^_*g*(*y*)_ is the genetic variance components for corresponding traits or VOL at 16 years.

Genetic gain (ΔG, %) was estimated using the correlated genetic gain equation [26]:

11 $$ \varDelta G\left(\%\right)=i{\displaystyle {h}_i^2}\sqrt{{\displaystyle {\sigma}_p^2}}/\overline{X}\times 100\% $$

Where *i* is selection intensity (*i* is equal to 2.064 when the selection ratio is 5 % and is 1.755 when the selection ratio is 10 %), *h*_*i*_^2^ is individual heritability, σ^2^_p_ is phenotypic variance and $\overline{\mathrm{X}}$ is the overall mean of growth traits.

The breeding values of individuals were estimated using the method of Best Linear Unbiased Prediction (BLUP) [27].

The measured growth data of 78 common families at age 1, 2, 4–7 and 16 in two sites were analyzed for family by site interaction. An orchard analysis indicated that the orchard effects were negligible. The Eq. (12) was used to estimate family by site interactions analysis:

12 $$ {Y}_{ijk\mathrm{l}}=\mu + {\mathrm{S}}_i+\mathrm{B}{\left(\mathrm{S}\right)}_{ij} + {\mathrm{f}}_{\mathrm{k}} + {\mathrm{f}\mathrm{s}}_{i\mathrm{k}} + \mathrm{f}\mathrm{b}{\left(\mathrm{s}\right)}_{ij\mathrm{k}}+{\mathrm{w}}_{ijkl} $$

Where *Y*_*ijk*l_ is the observation on the *l*^*th*^ tree in the *k*^*th*^ family in *j*^*th*^ block of the *i*^*th*^ site, *μ* is the overall mean, S_*i*_ is the fixed effect of *i*^*th*^ site, B(S)_*ij*_ is the fixed effect of the *j*^*th*^ block within the *i*^*th*^ site, f_k_ is the random effect of *k*^*th*^ family which follows the NID(0, *σ*_*f*_^2^), fb(s)_*ij*k_ is the random interaction effect of the *k*^*th*^ family by the *i*^*th*^ site which follows the NID(0, *σ*_*fs*_^2^), fb(*s*)_*ij*k_ is the random interaction effect of the *k*^*th*^ family by the j block within the *i*^*th*^ site which follows the NID (0, *σ*_*fb*(*s*)_^2^) and the w_*ijkl*_ is the residual which follows the NID (0, *σ*_*e*_^2^).

Family by site interactions were also assessed by estimating type-B genetic correlations between sites [28], expressed as

13 $$ rB={\sigma_f}^2/\left({\sigma_f}^2+{\sigma_{sf}}^2\right) $$

Where *σ*_*f*_^2^ and *σ*_*sf*_^2^ respectively represent the variance components of family and family by site interaction. It means no interaction when *rB* is close to 1.0 and significant rank changes among genotypes from one location to another when *rB* is small [29]. The interaction was considered not important if the estimate of *rB* was more than 0.67 [30].

The mixed linear model (1) was used to estimate the breeding values of families, which were obtained from the random effects of family. Spearman correlation analysis and Kendall's Tau correlation analysis with two-tailed test (α = 0.01 or 0.05) were used for the rank correlations of family breeding values.

All the analyses mentioned above were performed using the ASReml-R [22].

## Results

### The growth performance of different families in two progeny trials

**Fig. 1:**
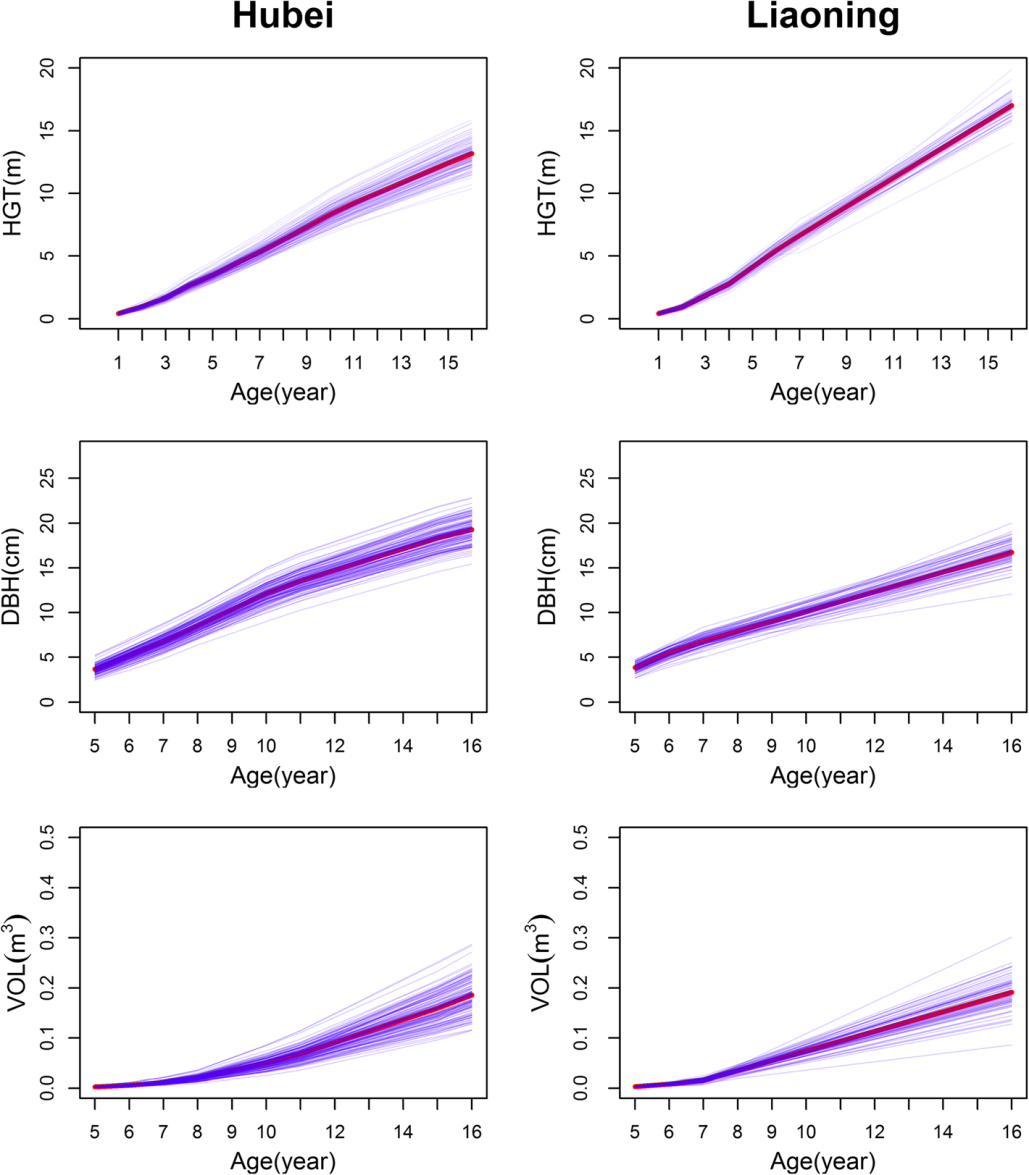
Growth trajectories of different families grown in Hubei and Liaoning. The thin lines with blue color represent the growth performance of different families and the thick lines with red color represent the growth performance on the whole

Stem growth was found to perform differently between two trials (Fig. 1). In general, families planted in Liaoning have greater height growth, but small diameter growth than those in Hubei, suggesting that trees are more slender in the former trial than in the latter trial. A larger variability was observed among families in Hubei than Liaoning. It is interesting to see that the form of growth is not only different between two trials, but also among families. All these implicate that different stem growth traits may result from distinct patterns of genetic control, also depending on environmental factors. In Liaoning and Hubei, the four controls from China performed better than partial families introduced from Japan, but worse than the others.

### Variance components and heritability estimates

The variance components, CVG and CVP for growth traits were shown in Table 3. There were significant differences on HGT, DBH and VOL among families at each year. The age trends of variance components were different in Hubei and Liaoning. In Hubei, the *σ*_*f*_^2^ and *σ*_*e*_^2^ for HGT, DBH and VOL and the *σ*_*fb*_^2^ for HGT and VOL showed an increased trend with time. However the *σ*_*fb*_^2^ for DBH increased with age, peaking at age 10 and decreased after. In Liaoning, the *σ*_*fb*_^2^ and *σ*_*e*_^2^ for HGT, DBH and VOL and *σ*_*f*_^2^ for DBH and VOL increased with time on the whole. However, the *σ*_*f*_^2^ for HGT increased at early years basically, but declined at age 16. The *σ*_*e*_^2^ was much larger than the *σ*_*f*_^2^ and *σ*_*fb*_^2^ in these two trials. Age trends of CVP and CVG in these two progeny trials presented certain rules. In Hubei, the CVP and CVG for HGT, DBH and VOL decreased at early years and kept relatively stable later. In Liaoning, both the CVP and CVG for HGT, DBH and VOL showed a declined trend at all measured years. In these two trials, CVP and CVG for VOL were always higher than those for HGT and DBH at each year, and CVP for DBH were always higher than those for HGT.

**Table 3:** The variance components with standard error (SE) in parentheses and variation coefficients for growth traits at different age in Hubei and Liaoning (*σ*_*f*_^2^, *σ*_*e*_^2^ and *σ*_*e*_^2^ represent the variance components of family, family by block interaction and residual error respectively; CVG and CVP stand for genetic variation coefficients and phenotypic variation coefficient; Site1 stands for Hubei and Site2 stands for Liaoning)

Age	σ_f_^2^		σ_fb_^2^		σ_e_^2^		CVG (%)		CVP (%)	
	Site1	Site2	Site1	Site2	Site1	Site2	Site1	Site2	Site1	Site2
h1	1.51E-03^***^(5.83E-04)	5.10E-03^***^(1.13E-03)	7.47E-03^***^(9.61E-04)	3.62E-03^***^(7.14E-04)	3.80E-02(1.04E-03)	1.59E-02(7.00E-04)	9.30	16.07	51.85	35.31
h2	3.52E-03^***^(1.81E-03)	1.06E-02^***^(3.20E-03)	3.27E-02^***^(3.33E-03)	1.73E-02^***^(3.42E-03)	9.13E-02(2.65E-03)	8.01E-02(3.50E-03)	6.04	10.63	36.36	33.92
h3	1.04E-02^***^(3.72E-03)		5.80E-02^***^(5.87E-03)		1.89E-01(5.02E-03)		6.09		30.27	
h4	2.67E-02^***^(7.93E-03)	2.56E-02^***^(1.12E-02)	1.29E-01^***^(1.15E-02)	8.99E-02^***^(1.65E-02)	2.07E-01(7.02E-03)	3.58E-01(1.57E-02)	6.18	5.72	22.82	24.61
h5	4.77E-02^***^(1.19E-02)	4.02E-02^***^(1.73E-02)	1.63E-01^***^(1.46E-02)	1.40E-01^***^(2.48E-02)	3.83E-01(1.02E-02)	5.21E-01(2.28E-02)	6.29	4.82	22.19	20.12
h6	7.31E-02^***^(1.76E-02)	3.65E-02^***^(2.06E-02)	2.26E-01^***^(2.07E-02)	1.78E-01^***^(3.37E-02)	5.77E-01(1.53E-02)	7.40E-01(3.25E-02)	6.15	3.48	21.27	17.82
h7	1.11E-01^***^(2.52E-02)	4.72E-02^***^(2.80E-02)	2.97E-01^***^(2.78E-02)	2.33E-01^***^(4.57E-02)	7.97E-01(2.12E-02)	1.06E + 00(4.61E-02)	6.27	3.24	20.61	17.22
h8	1.56E-01^***^(3.38E-02)		3.70E-01^***^(3.54E-02)		1.04E + 00(2.78E-02)		6.27		19.87	
h10	2.93E-01^***^(5.92E-02)		5.89E-01^***^(5.85E-02)		1.27E + 00(4.29E-02)		6.49		17.57	
h11	3.52E-01^***^(6.99E-02)		6.49E-01^***^(6.73E-02)		1.57E + 00(5.25E-02)		6.44		17.39	
h15	6.10E-01^***^(1.18E-01)		8.56E-01^***^(1.10E-01)		3.17E + 00(1.07E-01)		6.28		17.32	
h16	6.55E-01^***^(1.29E-01)	1.33E-02^**^ (6.65E-02)	9.22E-01^***^(1.25E-01)	6.85E-01^***^(1.44E-01)	3.71E + 00(1.25E-01)	2.02E + 00(1.23E-01)	6.14	0.68	17.43	9.67
b5	1.24E-01^***^(3.21E-02)	7.62E-02^***^(3.56E-02)	3.84E-01^***^(4.04E-02)	2.81E-01^***^(5.34E-02)	1.37E + 00(3.65E-02)	1.22E + 00(5.35E-02)	9.64	7.1	37.62	32.32
b6	1.90E-01^***^(4.78E-02)	7.44E-02^***^(4.24E-02)	5.65E-01^***^(5.90E-02)	2.70E-01^***^(6.99E-02)	1.99E + 00(5.25E-02)	1.98E + 00(8.61E-02)	8.35	4.92	31.76	27.5
b7	2.67E-01^***^(6.51E-02)	1.08E-01^***^(5.58E-02)	6.97E-01^***^(7.80E-02)	2.72E-01^***^(8.68E-02)	2.84E + 00(7.52E-02)	2.70E + 00(1.18E-01)	7.61	4.76	28.72	25.46
b8	3.23E-01^***^(7.98E-02)		8.83E-01^***^(9.74E-02)		3.42E + 00(9.13E-02)		6.66		25.23	
b10	5.63E-01^***^(1.32E-01)		1.25E + 00^***^(1.59E-01)		4.44E + 00(1.50E-01)		6.19		20.62	
b11	6.52E-01^***^(1.49E-01)		1.24E + 00^***^(1.76E-01)		5.40E + 00(1.81E-01)		5.95		19.90	
b15	1.03E + 00^***^(2.26E-01)		9.48E-01^***^(2.60E-01)		1.07E + 01(3.59E-01)		5.54		19.42	
b16	1.12E + 00^***^(2.45E-01)	2.58E-01^*^ (1.89E-01)	7.93E-01^**^ (2.84E-01)	6.76E-01^*^ (3.50E-01)	1.23E + 01(4.16E-01)	7.23E + 00(4.37E-01)	5.48	3.02	19.56	17.00
v5	3.59E-07^***^(8.34E-08)	3.79E-07^***^(1.59E-07)	8.74E-07^***^(9.35E-08)	1.20E-06^***^(2.18E-07)	3.34E-06(8.86E-08)	5.01E-06(2.18E-07)	23.25	16.87	83.02	70.33
v6	1.60E-06^***^(3.58E-07)	1.11E-06^***^(5.52E-07)	3.67E-06^***^(3.87E-07)	3.58E-06^***^(8.40E-07)	1.36E-05(3.58E-07)	2.29E-05(9.92E-07)	21.02	11.98	72.18	59.75
v7	5.13E-06^***^(1.12E-06)	3.38E-06^***^(1.57E-06)	1.04E-05^***^(1.17E-06)	8.41E-06^***^(2.27E-06)	4.38E-05(1.16E-06)	6.62E-05(2.87E-06)	19.55	11.62	66.48	55.81
v8	1.36E-05^***^(2.91E-06)		2.64E-05^***^(2.97E-06)		1.10E-04(2.92E-06)		18.14		60.21	
v10	7.48E-05^***^(1.52E-05)		1.16E-04^***^(1.51E-05)		4.40E-04(1.49E-05)		17.32		50.30	
v11	1.28E-04^***^(2.56E-05)		1.75E-04^***^(2.50E-05)		7.87E-04(2.63E-05)		16.71		48.75	
v15	6.04E-04^***^(1.16E-04)		4.35E-04^***^(1.11E-04)		4.46E-03(1.49E-04)		15.44		46.59	
v16	7.83E-04^***^(1.52E-04)	1.14E-04^*^ (1.11E-04)	4.72E-04^***^(1.47E-04)	5.26E-04^**^ (2.15E-04)	6.19E-03(2.08E-04)	4.16E-03(2.52E-04)	15.08	5.56	46.49	36.04

^***^ = significant at 0.001 level, ^**^ = significant at 0.01 level, ^*^ = significant at 0.05 level

**Fig. 2:**
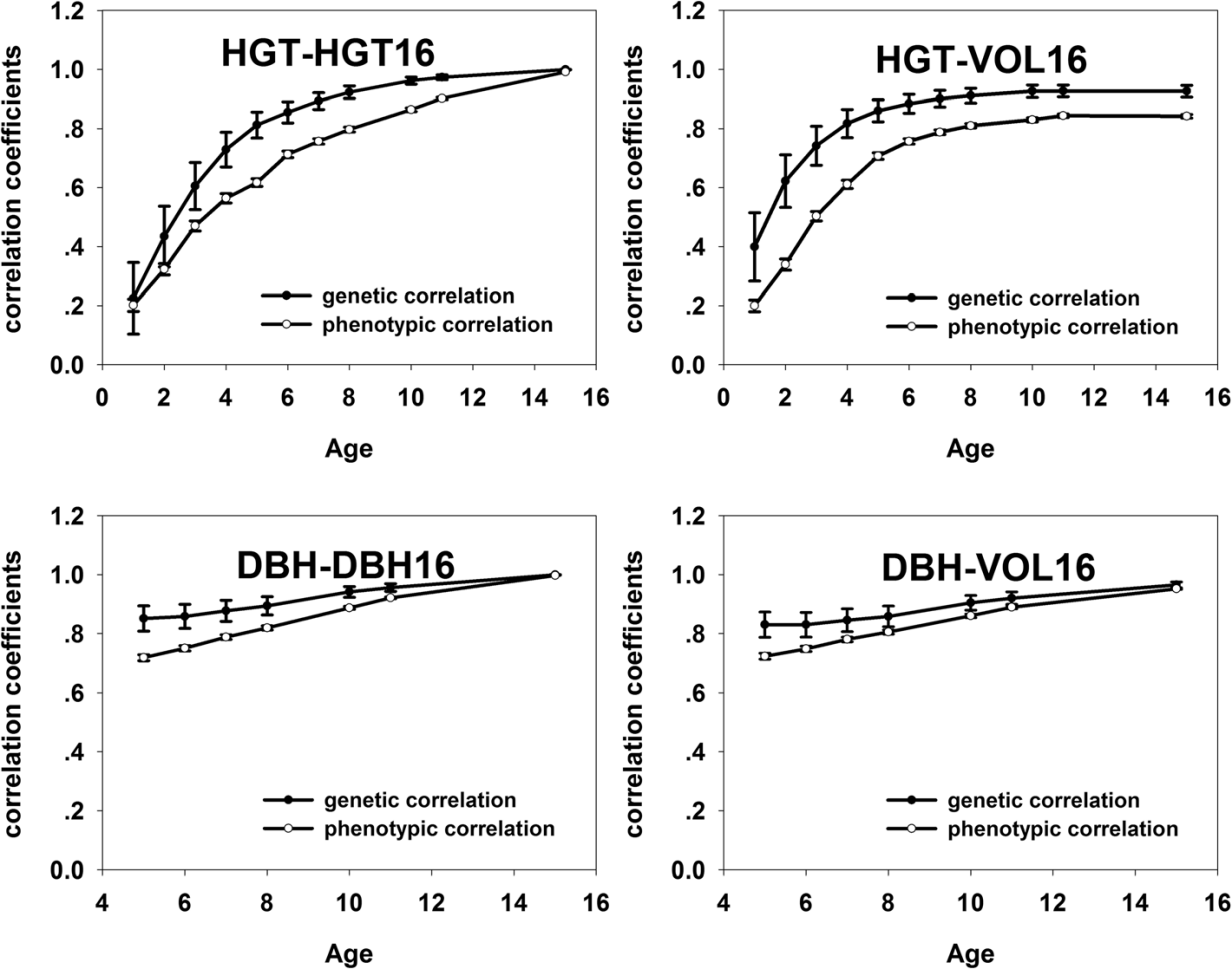
The age trends of heritability estimates for growth traits in Hubei. The figures represent the age trends of individual-tree and family heritability estimates for HGT, DBH and VOL respectively in Hubei. The lines with hollow circles represent the family heritability estimates and the lines with solid circles represent the individual heritability estimates and the bars stands for standard errors

The individual heritability estimates (*h*_*i*_^2^) and family heritability estimates (*h*_*f*_^2^) for HGT, DBH and VOL also showed different time trends in Hubei and Liaoning (Fig. 2, Additional file 1: Table S1). The *h*_*i*_^2^ and *h*_*f*_^2^ for HGT in Hubei increased at early years and kept in the narrow range of 0.50–0.55 and 0.63–0.66 after age 10. However, the *h*_*i*_^2^ and *h*_*f*_^2^ for HGT in Liaoning decreased sharply with time in the range of 0.02–0.83 and 0.05–0.77. The *h*_*i*_^2^ and *h*_*f*_^2^ for DBH and VOL were relatively stable in these two trials. In Hubei, *h*_*i*_^2^ and *h*_*f*_^2^ for DBH ranged from 0.26 to 0.36 and 0.49 to 0.58 and those for VOL ranged from 0.31 to 0.47 and 0.55 to 0.65. In Liaoning, *h*_*i*_^2^ and *h*_*f*_^2^ for DBH were in the range of 0.13–0.19 and 0.29–0.39, and those for VOL were in the range of 0.10–0.23 and 0.23–0.44. The *h*_*i*_^2^ and *h*_*f*_^2^ for DBH and VOL in Hubei were larger than those in Liaoning. In Hubei, *h*_*i*_^2^ and *h*_*f*_^2^ for HGT were higher than those for DBH and *h*_*f*_^2^ for HGT, DBH and VOL were more than 0.49 from age 5 to 16.

### The age-age genetic and phenotypic correlations

#### Hubei trial

**Fig. 3:**
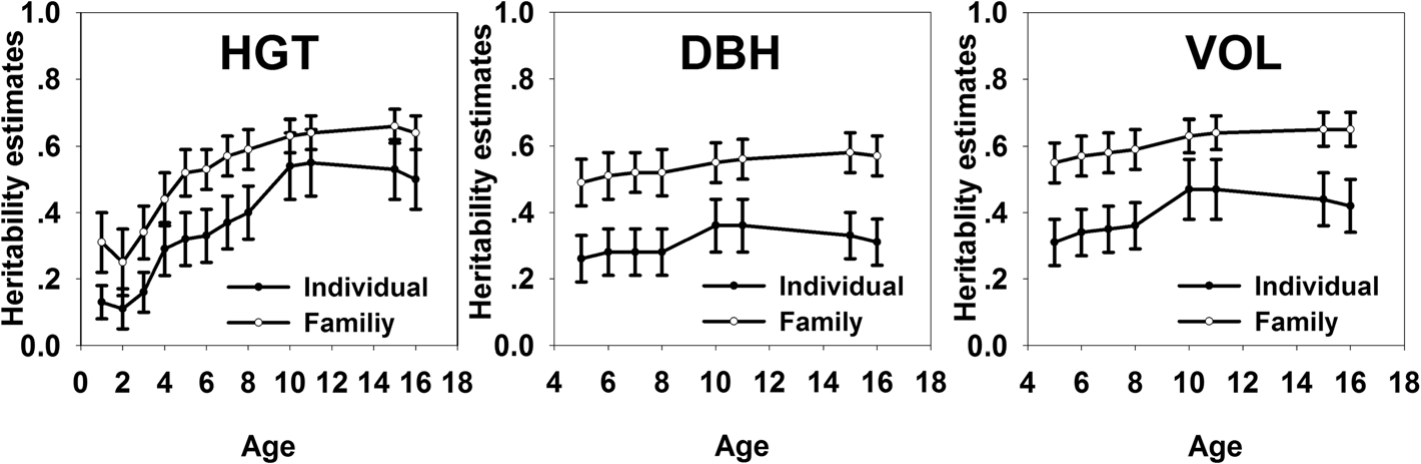
Age-age related genetic and phenotypic correlations in Hubei. HGT-HGT16 and DBH-DBH16 represent age-age related genetic correlations and phenotypic correlations between HGT and DBH at early ages and corresponding growth traits at 16 years. HGT-VOL16 and DBH-VOL 16 represent age-age related genetic correlations and phenotypic correlations between HGT and DBH at early ages and VOL at 16 years. The lines with hollow circles represent the phenotypic correlations and the lines with solid circles represent the genetic correlations and the bars stands for standard errors

The age-age genetic and phenotypic correlations between growth traits (HGT or DBH) at early ages and corresponding growth traits or VOL at age 16 increased over time (Fig. 3, Additional file 2: Table S2). The estimated genetic correlations were mostly more than the corresponding estimated phenotypic correlations. The estimated genetic correlations for HGT-HGT16 varied widely from 0.225 to 0.999 and the corresponding estimated phenotypic correlations varied from 0.202 to 0.991. The estimated phenotypic and genetic correlations for HGT-HGT16 were more than 0.5 at age 4. The estimated phenotypic correlation and genetic correlations for DBH-DBH16 ranged from 0.851 to 0.999 and 0.718 to 0.997 and both of them exceeded 0.5 at age 5. The age-age genetic and phenotypic correlations between HGT at early ages and VOL at 16 years were in the range of 0.399–0.926 and 0.200–0.842, in which the genetic correlation were more than 0.5 at age 2 and phenotypic correlations were more than 0.5 at age 3. Those for DBH-VOL16 ranged from 0.830 to 0.965 and 0.723 to 0.952, both of which were more than 0.5 at age five.

The age-age genetic correlation for DBH-DBH was stronger than that of HGT-HGT at five and six years and after that those for HGT-HGT were stronger than DBH-DBH. At young ages (<11 years), HGT was more genetically correlated to VOL than DBH, and at age 15 HGT was less correlated to final VOL than DBH. At the phenotypic level, HGT was less correlated to VOL than DBH at age five.

#### Liaoning trial

The age-age genetic correlations for HGT-HGT16 and HGT-VOL 16 were in the range of 0.182–0.716 and 0.425–0.701 and both of them exceeded 0.5 at age four, which showed basically upward trend except the ones with little decrease at age six and seven. The age-age phenotypic correlations for HGT-HGT16 and HGT-VOL 16 increased over time ranging from 0.162 to 0.735 and 0.247 to 0.734 and both of them were more than 0.5 at age 4. The age-age genetic and phenotypic correlations for DBH-DBH16, DBH-VOL16 were relatively stable. Those for DBH-DBH16 ranged from 0.689 to 0.723 and 0.658 to 0.780 and those for DBH-VOL16 ranged from 0.674 to 0.706 and 0.681 to 0.791, which were all above 0.5. The age-age genetic correlations and phenotypic correlations for DBH-DBH were a little stronger than that of HGT-HGT at age five and six. At age five, HGT was more genetically correlated to VOL than DBH, but was less correlated to VOL than DBH at phenotypic level.

### Family by site interaction

The type-B genetic correlations (r_B_) and rank correlations of family breeding value for HGT, DBH and VOL showed the similar trends over time (Table 4) on the whole. The r_B_ for HGT, DBH and VOL were less than 0.6 at age 1, 2, 4, 5, 6 and 7 and were 0.50, 0.60 and 0.65 at age 16 respectively. The spearman rank correlations coefficients of family breeding value for HGT, DBH and VOL were less than 0.3 at age 1, 2, 4, 5, 6 and 7 and the ones at age 16 were 0.35, 0.24 and 0.33 respectively. The Kendall's Tau correlation coefficients were all less than 0.3 at age 1, 2, 4, 5, 6, 7 and 16. Comparing the breeding values rankings of volume at age 16, there were no common families at the top ten percent of rankings at the two sites and the number of common families were 5, 10, 16 and 20 when the top ratios of rankings were 20, 30, 40, 50 % respectively. All the families ranked differently from one site to another. Family by site interaction was significant for VOL based on the type-B genetic correlations and rank correlations analysis, which is focused on in breeding programs.

**Table 4:** Type-B genetic correlations with standard error (SE) in parentheses and spearman rank correlations coefficients of family breeding values with Kendall's Tau correlation coefficients in parentheses across Hubei and Liaoning

	Type-B genetic correlations			Rank correlations of family breeding values		
Age	HGT	DBH	VOL	HGT	DBH	VOL
1	0.27 (0.15)			0.23^*^ (0.17^*^)		
2	0.22 (0.17)			0.13 (0.08)		
4	0.27 (0.18)			0.18 (0.08)		
5	0.40 (0.15)	0.27 (0.18)	0.25 (0.17)	0.27^*^ (0.19^*^)	0.15 (0.11)	0.20 (0.15)
6	0.40 (0.16)	0.41 (0.18)	0.36 (0.17)	0.26^*^ (0.18^*^)	0.20 (0.14)	0.23^*^ (0.16^*^)
7	0.34 (0.16)	0.38 (0.18)	0.33 (0.18)	0.25^*^ (0.17^*^)	0.16 (0.12)	0.24^*^ (0.16^*^)
16	0.50 (0.16)	0.60 (0.23)	0.65 (0.18)	0.35^*^ (0.23^*^)	0.24^*^ (0.17)	0.33^**^ (0.24^**^)

^**^ = significant at 0.01 level, ^*^ = significant at 0.05 level

### Genetic gain and early selection

We determined the optimum selection age for HGT (or DBH) when the age-age genetic and phenotypic correlations for HGT-HGT16 (or DBH-DBH16) and HGT-VOL16 (or DBH-VOL16) were more than 0.5. The phenotypic and genetic correlations for HGT-HGT16 and HGT-VOL16 exceeded 0.5 at age four and those for DBH-DBH16 and DBH-VOL16 were more than 0.5 at age five in Hubei and Liaoning. Taking volume at age 16 as target traits, the optimum selection age decided in our research were at age four for HGT and five for DBH. In Hubei, the genetic gains for VOL were 40.4 and 34.4 % in individual selection at age 16 when the selection ratio was 5 and 10 %. The corresponding genetic gains for VOL in Liaoning were 7.07 and 6.04 %. Taking genetic gains and genetic diversity into consideration, 10 % was the appropriate selection ratio for selection of advanced breeding population.

The effect of early selection was confirmed through comparison of consistency between early selection at different selection ratios and terminal selection at age 16 at 10 % selection ratio according to the breeding values rank. The numbers how many individuals in the early selection were consistent with the top 10 % of total individuals at age 16 and the successful ratios were listed in Table 5. In Hubei, the successful ratios for D5-V16 were higher than those for H4-V16 at each early selection ratio. The successful ratios for H4-V16 was more than 50 % when the early selection ratios was 20 % and the successful ratios for D5-V16 was more than 50 % when the early selection ratios was 10 %. The successful ratios for early selection in Liaoning were much lower than those in Hubei. However, the successful ratios for H4-V16 and D5-V16 were more than 50 % when the early selection ratio was 20 %. These indicate that the early selection at four years for HGT and five years for DBH was a very effective way for breeding program of *L.kaempferi* in Hubei and Liaoning.

**Table 5:** Comparison of consistency between early selection and selection at age 16 at 10 % selection ratio (H4 stands for selection at age four based on the HGT as selection criterion; D5 stands for selection at age five based on the DBH as selection criterion; V16 represents for selection at age 16 taking VOL as target traits

	Hubei				Liaoning			
	H4-V16		D5-V16		H4-V16		D5-V16	
Early selection ratio	Numbers	Success ratio	Numbers	Success ratio	Numbers	Success ratio	Numbers	Success ratio
		(%)		(%)		(%)		(%)
10 %	121	48.79	159	64.11	40	28.57	45	32.14
20 %	165	66.53	199	80.24	74	52.86	72	51.43
30 %	194	78.23	210	84.68	96	68.57	101	72.14
40 %	214	86.29	229	92.34	109	77.86	116	82.86
50 %	224	90.32	236	95.16	123	87.86	127	90.71

## Discussion

Although few studies about early selection of *L. kaempferi* in China have been reported, more precise and stable estimates of genetic parameters were needed to predict optimum early selection age. Regionalization deployment is an important strategy in tree breeding program, however studies about genotype by environment interactions for *L. kaempferi* in China were seldom reported. Two progeny trials established in Hubei and Liaoning in our research provide rich genetic sources to research on these issues.

We observed the different performance among families from the growth trajectories indicating the huge potential for genetic improvement in *L. kaempferi* breeding programs, which is supported by the analysis of variance components that there are significant differences on HGT, DBH and VOL among families at each age.

Age trends of variance components, CVG and CVP for growth traits present certain rules with some difference between Hubei and Liaoning. The phenomenon that the *σ*_*e*_^2^ was much larger than the *σ*_*f*_^2^ and *σ*_*fb*_^2^ might be result of some components which is not separated from environmental factor or some other unknown reasons in agreement with Sun [31]. The CVG and CVP for VOL were higher than those for DBH and HGT, which is in agreement with the study by Cornelius et al. [32] and Lai et al. [4]. Age trends of CVP, to some extent, presented the trends of completion among individuals [31]. The fact that CVP for DBH was always higher than that for HGT indicates that DBH was more sensitive to competition than HGT supported by some other studies [33, 34]. CVG, that is, the genetic variance standardized to trait mean, is used to measure the extent of genetic variation present in a population and less additive genetic variation was predicted to be closely related to fitness [35]. The CVG and CVP kept stable in Hubei trial after age 8, but there was a sharp decrease at age 16 in Liaoning trial, which might be due to that thinning at age 14 without tree breeder’s guide reduced the diversity in populations.

The heritability reflects the degree of genetic control for growth traits. Two progeny trials performed differently in heritability estimates for growth traits, which is supported by other studies [36, 37]. The fact that the *h*_*i*_^2^ and *h*_*f*_^2^ for HGT kept in the range of 0.29–0.55 and 0.44–0.66 after 4 years in Hubei indicates that it was relatively reliable to make the early selection after age 4. However, *h*_*i*_^2^ and *h*_*f*_^2^ for HGT decreased deeply in Liaoning progeny trial from early age to 16 similar to the study by Sun [6] who found that the age trends of heritability estimates decreased for HGT of *L. kaemperi* in Hubei from 5 to 12 years. The *h*_*i*_^2^ and *h*_*f*_^2^ in Liaoning at age one is abnormal. This might be due to unstable performance of seedlings after the transplant from Hubei to Liaoning. The very low estimates at age 16 might be the result of relatively large residual error of the experiment or low genetic variation. The heritability estimates for DBH and VOL in these two progeny trials were relatively stable similar to the studies by Xiang [38], which indicate that DBH might be a better predictor in early selection in Hubei and Liaoning. The heritability estimates for DBH and VOL in Hubei trial were higher than those in Liaoning trial which might be result of different family sizes or geographical locations. A study in Norway spruce reveals that *h*_*i*_^2^ for HGT have a tendency to decrease with test-site latitude [39]. Therefore, we speculated that latitude might be one of the reasons that the *h*_*i*_^2^ and *h*_*f*_^2^ for DBH and VOL in Liaoning were lower than those in Hubei. In Hubei, the *h*_*i*_^2^ and *h*_*f*_^2^ for DBH were lower than those for HGT in agreement with some other studies [40, 41]. These heritability estimates for growth traits were important for the breeding programs.

Treating volume as the target traits, dual growth traits selection was recommended in our research. For many programs, the best early predictor and selection trait for volume yield at rotation has been HGT due to its higher heritability and juvenile-mature correlation, as well as less effect caused by spacing which result from mortality and thinning than other traits [42–44]. Whereas others have indicated that DBH would be more efficient for early selection [45]. Our research indicates that dual growth traits selection for *L.kaempferi* was more reliable than single growth trait selection in early selection, in agreement with the results observed by Lai [4]. In Hubei, HGT was more genetically correlated to VOL than DBH at young ages (<11 years) in agreement with Gwaze and Bridgewater who reveals that HGT at young age (< seven years) was more genetically correlated to VOL at 25 years [41]. However, the successful ratios of early selection taking DBH at age five as selection criterion were higher than that taking HGT at age four as selection criterion. In Liaoning, the *h*_*i*_^2^ and *h*_*f*_^2^ for DBH progeny trial kept relatively stable, which indicates that DBH should be a better predictor in early selection. However, HGT was more genetically correlated to VOL than DBH at age five. Meanwhile, the successful ratios were very close taking HGT and DBH as criterion when the early selection ratio was 20 %. Therefore dual growth traits selection was recommended in our research.

Recent studies proposed different optimum early selection age for *L.kaempferi*. Sun et al. [6] found that the optimum early selection age of *L.kaempferi* in Hubei was six years for HGT and DBH in family selection and eight years in individual-tree selection. Lai et al. [4] found that the optimum early selection age for 78 clones in Henan was five years for DBH, but corresponding early selection age for HGT was two years; Ma found the optimum age of early selection was 10 years for HGT in 10 clones of *L. kaempferi* in Liaoning [7]. These different determinations might be due to the different experiment designs, materials, sites and the numbers of materials. The optimum early selection ages determined in our research were four for HGT and five for DBH in Hubei and Liaoning. Despite the limitations of number of families and long investigation interval in Liaoning, high early selection successful ratios were achieved. More precise parameter estimates based on larger populations in Hubei made the determination of early selection age more reliable, which is very helpful for breeding programs. Summary of these studies including Hubei, Henan and Laioning across two climate zones, the optimum early selection ages for DBH were relatively stable, which might provide important reference to breeding programs in these provinces and the surrounded places. *L.kaempferi* trees can be propagated vegetatively and induced to flower in early time for hybridization, so that early selection can short the breeding cycle in next generation breeding programs, which can remain the epistatic effects and the additive genetic effect. Now there have been a seed orchards constructed in Hubei. These two progeny trials in our research are important germplasm resources of *L.kaempferi* in China. The major rotation age for *L. kaempferi* is about 25–28 years as pulpwood in China [4]. The growth trend has not reached a plateau at age 16 in our research and no longer collection data can be used. Although the successful ratios of the early selection were very high, more accurate estimates with longer collected data should be done in future.

*L. kaempferi* is distributed widely in China, including northeast of China, Henan, Hebei, Shandong, Hubei, Jiangxi, Sichuan and so forth, so that the environment factors must be considered in *L. kaempferi* breeding programs to maximize long-term genetic gain. The different annual increment for HGT and DBH across Hubei and Liaoning showed phenotypic plastic of *L. kaempferi* in adapting different environments. Although we cannot observe significant difference from the growth trajectories of VOL, type-B correlations and rank correlations of family breeding value confirm that there are significant family-by-site interactions for *L. kaempferi* across Hubei and Liaoning. Thus it might be a wise choice for tree breeders to develop populations specifically adapted to each environment to maximize genetic gain for *L. kaempferi* in *China,* which was in accordance with previous studies in Japan [19, 20]. However, it is difficult to determine the necessity of regionalization deployment for *L. kaempferi* in China in our research due to the fact that two sites in our research were so limited and economic costs were not estimated. Moreover both the r_B_ and the rank correlations of family breeding value showed a basically upward trends and reached the maximum at age 16 (r_B_ =0.65) indicating that these correlations might increase in the next years. We cannot speculate the family by site interaction in the next years would increase or decrease and further studies should be carried out in the future.

## Conclusions

There are significant differences on growth traits among families at each year. The genetic parameters varied dynamically and present certain regularity. The genetic and phenotypic correlations between early age and mature age for growth traits increased with time and surpassed 0.5 at 4 or 5 year. Based on trends of genetic parameters and genetic correlations analysis, the optimum early selection age was 4 years for HGT and 5 years for DBH in Hubei and Liaoning. The dual trait selection was recommended in our research for early selection. Family-by-site analysis revealed that the effect of family by site interaction were significant in three growth traits. Regionalization deployment should be given more attention in tree breeding programs *of L. kaempferi*.

## Additional files

Additional file 1: The heritability estimates for growth traits in Hubei and Liaoning with standard error (SE) in parentheses. The *h*_*i*_^2^ stands for individual-tree heritability estimates and *h*_*f*_^2^ stands for family heritability estimates. (DOCX 39 kb) Additional file 2: Age-age related genetic and phenotypic correlations in Hubei and Liaoning with standard error (SE) in parentheses. HGT-HGT16 and DBH-DBH16 represent age-age related genetic correlations and phenotypic correlations between HGT and DBH at early ages and corresponding growth traits at 16 years. HGT-VOL16 and DBH-VOL 16 represent age-age related genetic correlations and phenotypic correlations between HGT and DBH at early ages and VOL at 16 years. (DOCX 29 kb) Additional file 3: The data sets collected in our research. Site represents the collection site, Fam stands for the families and Rep stands for the block within sites. The letter of h stands for the HGT and the letter of b stands for the DBH. The number after the letters of h and b stand for the age. (XLSX 621 kb)
