# Supplementary material for: Age trends of genetic parameters, early selection and family by site interactions for growth traits in Larix kaempferi open-pollinated families
Source: BMC Genet. 2016 Jul 7;17:104. doi: 10.1186/s12863-016-0400-7 (PMC4936286; doi:10.1186/s12863-016-0400-7)
Supplement: Additional file 1: — The heritability estimates for growth traits in Hubei and Liaoning with standard error (SE) in parentheses. The h i 2 stands for individual-tree heritability estimates and h f 2 stands for family heritability estimates. (DOCX 39 kb) [file 12863_2016_400_MOESM1_ESM.docx]

Table S1 The heritability estimates for growth traits in Hubei and Liaoning with standard error (SE) in parentheses. The $h_{i}^{2}$ stands for individual-tree heritability estimates and $h_{f}^{2}$hf^2^ stands for family heritability estimates.

|  | Hubei | | | | | | Liaoning | | | | | |
| --- | --- | --- | --- | --- | --- | --- | --- | --- | --- | --- | --- | --- |
|  | HGT | | DBH | | VOL | | HGT | | DBH | | VOL | |
| Age | $h_{i}^{2}$ | $h_{f}^{2}$ | $h_{i}^{2}$ | $h_{f}^{2}$ | $h_{i}^{2}$ | $h_{f}^{2}$ | $h_{i}^{2}$ | $h_{f}^{2}$ | $h_{i}^{2}$ | $h_{f}^{2}$ | $h_{i}^{2}$ | $h_{f}^{2}$ |
| 1 | 0.13(0.05) | 0.31(0.09) |  |  |  |  | 0.83(0.15) | 0.77(0.04) |  |  |  |  |
| 2 | 0.11(0.06) | 0.25(0.10) |  |  |  |  | 0.39(0.11) | 0.59(0.08) |  |  |  |  |
| 3 | 0.16(0.06) | 0.34(0.08) |  |  |  |  |  |  |  |  |  |  |
| 4 | 0.29(0.08) | 0.44(0.08) |  |  |  |  | 0.22(0.09) | 0.42(0.11) |  |  |  |  |
| 5 | 0.32(0.08) | 0.52(0.07) | 0.26(0.07) | 0.49(0.07) | 0.31(0.07) | 0.55(0.06) | 0.23(0.10) | 0.43(0.11) | 0.19(0.09) | 0.39(0.12) | 0.23(0.09) | 0.44(0.11) |
| 6 | 0.33(0.08) | 0.53(0.06) | 0.28(0.07) | 0.51(0.07) | 0.34(0.07) | 0.57(0.06) | 0.15(0.08) | 0.33(0.13) | 0.13(0.07) | 0.32(0.13) | 0.16(0.08) | 0.36(0.12) |
| 7 | 0.37(0.08) | 0.57(0.06) | 0.28(0.07) | 0.52(0.06) | 0.35(0.07) | 0.58(0.06) | 0.14(0.08) | 0.32(0.14) | 0.14(0.07) | 0.35(0.12) | 0.17(0.08) | 0.39(0.12) |
| 8 | 0.40(0.08) | 0.59(0.06) | 0.28(0.07) | 0.52(0.07) | 0.36(0.07) | 0.59(0.06) |  |  |  |  |  |  |
| 10 | 0.54(0.10) | 0.63(0.05) | 0.36(0.08) | 0.55(0.06) | 0.47(0.09) | 0.63(0.05) |  |  |  |  |  |  |
| 11 | 0.55(0.10) | 0.64(0.05) | 0.36(0.08) | 0.56(0.06) | 0.47(0.09) | 0.64(0.05) |  |  |  |  |  |  |
| 15 | 0.53(0.09) | 0.66(0.05) | 0.33(0.07) | 0.58(0.06) | 0.44(0.08) | 0.65(0.05) |  |  |  |  |  |  |
| 16 | 0.50(0.09) | 0.64(0.05) | 0.31(0.07) | 0.57(0.06) | 0.42(0.08) | 0.65(0.05) | 0.02(0.10) | 0.05(0.23) | 0.13(0.09) | 0.29(0.16) | 0.10(0.09) | 0.23(0.18) |
