# Supplementary material for: Age trends of genetic parameters, early selection and family by site interactions for growth traits in Larix kaempferi open-pollinated families
Source: BMC Genet. 2016 Jul 7;17:104. doi: 10.1186/s12863-016-0400-7 (PMC4936286; doi:10.1186/s12863-016-0400-7)
Supplement: Additional file 2: — Age-age related genetic and phenotypic correlations in Hubei and Liaoning with standard error (SE) in parentheses. HGT-HGT16 and DBH-DBH16 represent age-age related genetic correlations and phenotypic correlations between HGT and DBH at early ages and corresponding growth traits at 16 years. HGT-VOL16 and DBH-VOL 16 represent age-age related genetic correlations and phenotypic correlations between HGT and DBH at early ages and VOL at 16 years. (DOCX 29 kb) [file 12863_2016_400_MOESM2_ESM.docx]

Table S2 Age-age related genetic and phenotypic correlations in Hubei and Liaoning with standard error (SE) in parentheses. HGT-HGT16 and DBH-DBH16 represent age-age related genetic correlations and phenotypic correlations between HGT and DBH at early ages and corresponding growth traits at 16 years. HGT-VOL16 and DBH-VOL 16 represent age-age related genetic correlations and phenotypic correlations between HGT and DBH at early ages and VOL at 16 years.

|  | Age | HGT-HGT16 | | HGT-VOL16 | | DBH-DBH16 | | DBH-VOL16 | |
| --- | --- | --- | --- | --- | --- | --- | --- | --- | --- |
|  |  |  |  |  |  |  |  |  |  |
| Hubei | 1 | 0.225(0.121) | 0.202(0.021) | 0.399(0.116) | 0.200(0.020) |  |  |  |  |
|  | 2 | 0.434(0.103) | 0.323(0.019) | 0.622(0.089) | 0.339(0.019) |  |  |  |  |
|  | 3 | 0.605(0.080) | 0.470(0.017) | 0.742(0.066) | 0.503(0.016) |  |  |  |  |
|  | 4 | 0.729(0.059) | 0.564(0.016) | 0.817(0.048) | 0.611(0.014) |  |  |  |  |
|  | 5 | 0.812(0.044) | 0.617(0.013) | 0.860(0.037) | 0.707(0.011) | 0.851(0.043) | 0.718(0.010) | 0.830(0.043) | 0.723(0.010) |
|  | 6 | 0.855(0.036) | 0.713(0.011) | 0.884(0.032) | 0.757(0.009) | 0.858(0.041) | 0.750(0.009) | 0.830(0.042) | 0.748(0.009) |
|  | 7 | 0.893(0.028) | 0.757(0.009) | 0.901(0.028) | 0.788(0.008) | 0.877(0.036) | 0.788(0.008) | 0.845(0.039) | 0.780(0.008) |
|  | 8 | 0.923(0.021) | 0.797(0.008) | 0.911(0.025) | 0.810(0.007) | 0.894(0.031) | 0.819(0.007) | 0.858(0.035) | 0.805(0.007) |
|  | 10 | 0.962(0.012) | 0.864(0.006) | 0.926(0.021) | 0.830(0.007) | 0.941(0.018) | 0.887(0.005) | 0.904(0.025) | 0.860(0.006) |
|  | 11 | 0.973(0.008) | 0.902(0.004) | 0.927(0.020) | 0.844(0.006) | 0.956(0.013) | 0.921(0.003) | 0.920(0.021) | 0.889(0.005) |
|  | 15 | 0.999(0.001) | 0.991(0.000) | 0.926(0.020) | 0.842(0.006) | 0.999(0.001) | 0.997(0.000) | 0.965(0.010) | 0.952(0.002) |
| Liaoning | 1 | 0.182(0.193) | 0.162(0.036) | 0.425(0.201) | 0.247(0.034) |  |  |  |  |
|  | 2 | 0.332(0.191) | 0.358(0.031) | 0.580(0.169) | 0.462(0.028) |  |  |  |  |
|  | 4 | 0.529(0.175) | 0.563(0.024) | 0.738(0.13) | 0.624(0.021) |  |  |  |  |
|  | 5 | 0.679(0.140) | 0.605(0.023) | 0.785(0.118) | 0.636(0.021) | 0.689(0.142) | 0.658(0.020) | 0.691(0.144) | 0.681(0.019) |
|  | 6 | 0.631(0.164) | 0.640(0.021) | 0.698(0.152) | 0.650(0.020) | 0.723(0.136) | 0.705(0.017) | 0.706(0.142) | 0.724(0.016) |
|  | 7 | 0.716(0.139) | 0.735(0.016) | 0.701(0.146) | 0.734(0.016) | 0.705(0.142) | 0.780(0.013) | 0.674(0.156) | 0.791(0.013) |
